# PD‐L1 (SP142) expression in neoplastic cells predicts a poor prognosis for patients with intravascular large B‐cell lymphoma treated with rituximab‐based multi‐agent chemotherapy

**DOI:** 10.1002/cam4.3104

**Published:** 2020-05-05

**Authors:** Yuka Suzuki, Kei Kohno, Kosei Matsue, Ayako Sakakibara, Eri Ishikawa, Satoko Shimada, Kazuyuki Shimada, Seiyo Mabuchi, Taishi Takahara, Seiichi Kato, Shigeo Nakamura, Akira Satou

**Affiliations:** ^1^ Department of Pathology and Laboratory Medicine Nagoya University Hospital Nagoya Japan; ^2^ Division of Haematology/Oncology Department of Internal Medicine Kameda Medical Centre Kamogawa Japan; ^3^ Department of Gastroenterology and Hepatology Nagoya University Graduate School of Medicine Nagoya Japan; ^4^ Department of Haematology and Oncology Nagoya University Graduate School of Medicine Nagoya Japan; ^5^ Department of Surgical Pathology Aichi Medical University Hospital Nagakute Japan; ^6^ Department of Pathology and Molecular Diagnostics Aichi Cancer Centre Hospital Nagoya Japan

**Keywords:** extranodal DLBCL, immunohistochemistry, intravascular large B‐cell lymphoma, PD‐L1, SP142

## Abstract

**Background:**

Intravascular large B‐cell lymphoma (IVLBCL) is a rare form of diffuse large B‐cell lymphoma (DLBCL) arising in extranodal sites. PD‐L1 expression of tumor cells has been reported in IVLBCL cells, but its clinicopathological relevance remains to be elucidated.

**Aims:**

This study was aimed to reveal the characteristics of PD‐L1^+^ IVLBCL.

**Methods and results:**

Neoplastic PD‐L1 expression was examined in 34 cases of IVLBCL and clinicopathological characteristics between patients with PD‐L1^+^ and PD‐L1^−^ IVLBCL were compared. We assessed PD‐L1 expression with SP142 antibody. Twelve (35%) of 34 cases showed positivity for PD‐L1. The PD‐L1^+^ group had significantly lower survival rates compared to the PD‐L1^−^ group. The PD‐L1^+^ IVLBCL group also had a significantly lower age distribution and a lower frequency of patients older than 60 years compared to the PD‐L1^−^ group. Very recently, we speculate that there is possible link between PD‐L1^+^ IVLBCL and PD‐L1^+^ extranodal DLBCL‐NOS (eDLBCL) because features of the two groups showed overlapping. Therefore, we compared the clinicopathological characteristics of the PD‐L1^+^ IVLBCL and PD‐L1^+^ eDLBCL. There were no significant differences in clinicopathological parameters and prognosis.

**Conclusion:**

The worse prognosis of the PD‐L1^+^ group might be caused by immune evasion mechanisms, which are linked to PD‐L1 expression. Therefore, PD‐L1^+^ IVLBCL cases might be regarded as good candidates for targeted immunotherapy. We also highlighted the overlapping features of PD‐L1^+^ IVLBCL and PD‐L1^+^ eDLBCL. This result suggests that they should be regarded as one entity, immune evasion‐related extranodal large B‐cell lymphoma.

## Graphical abstract

The PD‐L1^+^ intravascular large B‐cell lymphoma had significantly lower survival rates compared to the PD‐L1^−^ group. We speculate that the worse prognosis of the PD‐L1^+^ group was caused by immune evasion mechanisms, which are linked to PD‐L1 expression. Therefore, PD‐L1^+^ IVLBCL cases might be regarded as good candidates for targeted immunotherapy.

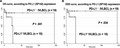

## 1 INTRODUCTION

Intravascular large B‐cell lymphoma (IVLBCL) is a rare form of diffuse large B‐cell lymphoma (DLBCL) arising in extranodal sites. IVLBCL is characterized by large tumor cells that proliferate predominantly, if not exclusively, in different‐sized blood vessels.^1^, ^2^, ^3^ IVLBCL is a systemic disease which potentially involve any organ, and it often disseminates widely.^2^, ^3^, ^4^ Currently, there is no good explanation for the heterogeneous clinical behaviors found among patients with IVLBCL. On the other hand, the new methods/technologies represented by incisional random skin biopsies,^5^ cell‐free DNA analyses in liquid biopsies,^6^ and rituximab‐based immunotherapy^7^ have dramatically advanced clinical approaches for patients with IVLBCL. Despite improved outcomes in IVLBCL, involvement and relapses in central nervous system (CNS) are serious complications, even in patients who receive multi‐agent chemotherapy with rituximab.^8^

Programmed death‐ligand 1 (PD‐L1) is an inhibitory immune check‐point molecule that suppresses the adaptive arm of the immune system. It is now well known that PD‐L1 promotes tumorigenesis by attenuating the activity of CD8^+^ T cells, which is specific to the tumor cells. This inhibition allows tumor cells to escape T cell‐mediated, tumor‐specific, and pathogen‐specific immune activities.^9^, ^10^ In recent studies, good results of immune check‐point inhibitors in treating malignant neoplasms, including lymphoma, have been reported.^11^, ^12^, ^13^, ^14^, ^15^, ^16^ In addition, PD‐L1 expression in tumor cells or microenvironmental cells might be a useful prognostic indicator.^17^, ^18^, ^19^, ^20^, ^21^ Immunohistochemical examinations of PD‐L1 have facilitated the diagnosis of lymphoid malignancies, mostly exemplified by classic Hodgkin lymphoma,^22^, ^23^ and they are useful for investigating potential therapeutic targets in relapsed/refractory lymphomas.^24^ PD‐L1 expression of tumor cells has been reported in IVLBCL.^17^ Very recently, Gupta et al investigated PD‐L1 expression in 11 cases of IVLBCL, and they evaluated the correlation between PD‐L1 expression and clinical and pathological features.^25^ That series showed that most clinical and pathological features were not significantly different between patients with PD‐L1^+^ and PD‐L1^−^ tumor cells. We very recently described overlapping features between PD‐L1^+^ IVLBCL and PD‐L1^+^ extranodal DLBCL‐NOS (eDLBCL).^26^ The PD‐L1^+^ eDLBCL cases showed frequent intravascular patterns, and all of them exclusively affected extranodal sites with no nodal lesions. Therefore, we suggested that PD‐L1^+^ IVLBCL and PD‐L1^+^ eDLBCL might be categorized into one proposed entity, immune evasion‐related extranodal large B‐cell lymphoma. However, the clinicopathological relevance of neoplastic PD‐L1 expression remains to be elucidated among patients with IVLBCL because the number of reported cases is limited.

This study aimed to reveal the characteristics of PD‐L1^+^ IVLBCL. To that end, neoplastic PD‐L1 expression in 34 cases of IVLBCL was investigated and the clinicopathological characteristics between patients with and without PD‐L1 positivity were compared.

## 2 MATERIAL AND METHODS

### 2.1 Patient samples

For this retrospective study, we retrieved data from our consultation files on 34 cases of IVLBCL, diagnosed between 2006 and 2018. Two cases were included in a previous report.^27^ The IVLBCL diagnosis was determined according to the 2017 WHO classification.^1^ All cases were independently reviewed by five pathologists to confirm the diagnosis and immunophenotype. All cases were negative for human immunodeficiency virus antibody by blood test.

The control group included seven patients previously diagnosed as PD‐L1^+^ extranodal DLBCL‐NOS (eDLBCL). The diagnosis of the seven cases was made between 2006 and 2018 in our hospital and these cases had been examined in a previous study.^26^, ^28^

All clinical and follow‐up data for the patients were obtained from medical records. Involved sites were determined by result of biopsy or radiographic evaluation, such as computed tomography or positron emission tomography. The institutional review board of our institution approved the study protocol.

### 2.2 Histological and immunohistochemical staining

Tissue samples were fixed in 10% formalin and embedded in paraffin. Then, 4‐μm thick sections were cut and stained with hematoxylin and eosin. Monoclonal antibodies against the following molecules were used for immunohistochemical study: L26/CD20, BCL‐2, BCL‐6 (DAKO), MUM‐1 (Santa Cruz Biotechnologies), CD3, CD5, CD10 (Novocastra Laboratories), and PD‐L1 (antibody SP142, Spring Bioscience). The antibodies were applied after sections were heated in a microwave oven for antigen retrieval. Tissue samples were considered positive for the expression of proteins, when more than 30% of tumor cells showed positive staining with a specific antibody.

### 2.3 Statistical analysis

Student's *t* test, Mann‐Whitney *U* test, χ^2^ test, and Fisher's exact test were used to assess the correlations between the two groups. The survival data of patients were analyzed with the Kaplan‐Meier method. The log‐rank test was used to test the difference in survival. Overall survival (OS) was calculated from the date of diagnosis to the date of death or last follow‐up. Disease‐specific survival (DSS) was calculated from the date of diagnosis to the date of disease‐specific death or last follow‐up. Progression‐free survival (PFS) was calculated from the date of diagnosis to the date of disease progression, first relapse, death from any cause, or the last follow‐up. All statistical analyses were performed with STATA software, version 12 (STATA Corporation).

## 3 RESULTS

### 3.1 Clinical characteristics of IVLBCL

Table 1 and Table S1 summarize the clinical characteristics of 34 patients with IVLBCL (16 males and 18 females; median age: 74 years, range: 51‐86). Diagnoses were established antemortem in 32 cases, and in two cases, at the time of autopsy. In the former group, the disease was diagnosed with histopathological examinations of skin (25 cases), skin and bone marrow (four cases), lung (one case), liver (one case), and bone marrow (one case) biopsies. The autopsy cases were documented separately.

**TABLE 1:** Clinical and phenotypic characteristics of patients with PD‐L1^+^ and PD‐L1^−^ IVLBCL

Variables	PD‐L1^+^ IVLBCL (n = 12)	PD‐L1^－^ IVLBCL (n = 22)	*P*
Sex (male/female)	5/7	11/11	.72
Age, median (range)	74 (51‐81)	75 (64‐86)	**.036**
Age > 60 y	9/12 (75%)	22/22 (100)	**.037**
Performance status > 1	8/12 (75%)	16/21 (76%)	.69
IPI (HI/H)	10/11 (91%)	20/21 (95%)	1.0
Stage III/IV	11/12 (92%)	21/21 (100%)	.36
plt < 14 × 10^4^/μL	9/11 (82%)	14/21 (67%)	.44
WBC < 3.5 × 10^3^/μL	4/11 (36%)	3/21 (14%)	.20
Alb < 3.0 g/dL	8/11 (73%)	19/21 (90)	.31
CRP > 1.0 mg/dL	11/11 (100%)	20/21 (95%)	1.0
sIL‐2R > normal	11/12 (92%)	21/21 (100%)	.36
LDH > normal	11/11 (100%)	20/21 (95%)	1.0
Hb < 11	9/11 (81%)	15/21 (71%)	.68
B symptoms	8/12 (67%)	19/22 (86%)	.21
Hepatomegaly	1/12 (8.3%)	4/19 (21%)	.62
Splenomegaly	7/12 (58%)	8/19 (42%)	.47
Respiratory symptoms	1/12 (8.3%)	10/18 (56%)	**.018**
CNS symptoms	6/12 (50%)	8/19 (42%)	.72
Cutaneous involvement	9/12 (75%)	16/22 (55%)	.61
Variant (Classic/HPS)	2/9	1/20	.27
CD5 positivity	2/7 (29%)	8/19 (42%)	.67
COO (GCB/non‐GCB subtype)	1/6	2/15	1.0

*P* value with siginificant difference are shown in bold value.

Abbreviations: Alb, albumin; CNS, central nervous system; COO, cell of origin; CRP, C‐reactive protein; GCB, germinal center B cell; H, high; Hb, hemoglobin; HI, high‐intermediate; HPS, hemophagocytic syndrome; IPI, international prognostic index; LDH, lactate dehydrogenase; plt, platelet; sIL‐2R, soluble interleukin‐2 receptor; WBC, white blood cell.

**FIGURE 1:**
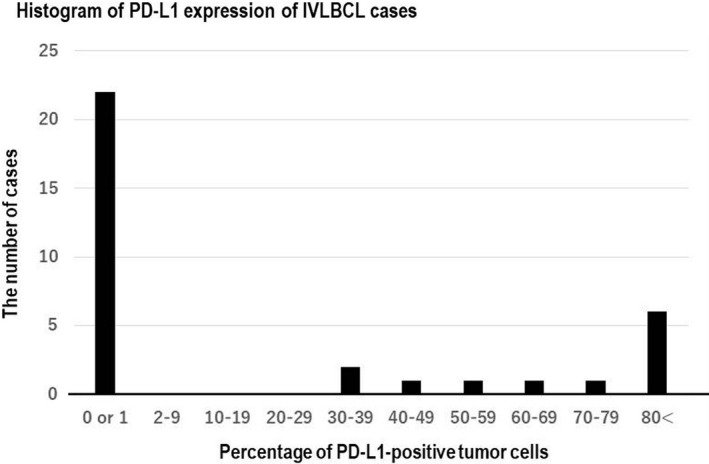
Histogram of PD‐L1 expression of intravascular large B‐cell lymphoma (IVLBCL) cases. The percentages of PD‐L1^+^ tumor cells of 12 cases ranged from 30% to 90%. On the other hand, those of the other 22 cases were 0% (n = 20) or 1% (n = 2)

Figure 1 is a histogram of PD‐L1 expression of IVLBCL cases. The percentages of PD‐L1^+^ tumor cells of 12 cases ranged from 30% to 90%. On the other hand, those of the other 22 cases were 0% (n = 20) or 1% (n = 2). Therefore, we used the cutoff value of 30% for PD‐L1 expression, and the 12 (35%) cases were considered positive for PD‐L1 (five males and seven females; median age: 74 years, range: 51‐81). Of these, we observed B symptoms (fever, weight loss, and night sweats) in eight (67%) patients, CNS symptoms in six (50%) patients, and respiratory symptoms in one (8.3%) patient. Moreover, of the patients with PD‐L1^+^ IVLBCL, seven (58%) had hepatosplenomegaly and 11 (92%) had IVLBCL stage III/IV. At presentation, laboratory data revealed that all patients tested (n = 11) had elevated lactate dehydrogenase (LDH) levels; nine (82%) had thrombocytopenia (platelet counts < 14 × 10^4^/μL), four (36%) had leukocytopenia (white blood cell [WBC] count < 3.5 × 10^3^/μL), and nine (81%) had anemia (hemoglobin < 11 g/dL). Based on the clinical findings, nine patients had the hemophagocytic syndrome (HPS) variant, and two had the classic form.

Compared to patients with PD‐L1^−^ IVLBCL, those with PD‐L1^+^ IVLBCL were significantly younger (*P* = .036) and a smaller proportion were older than 60 years (*P* = .037). Also, respiratory symptoms occurred significantly less frequently in the PD‐L1^+^ group than in the PD‐L1^−^ group (*P* = .018).

### 3.2 Histological and immunophenotypic characteristics

**FIGURE 2:**
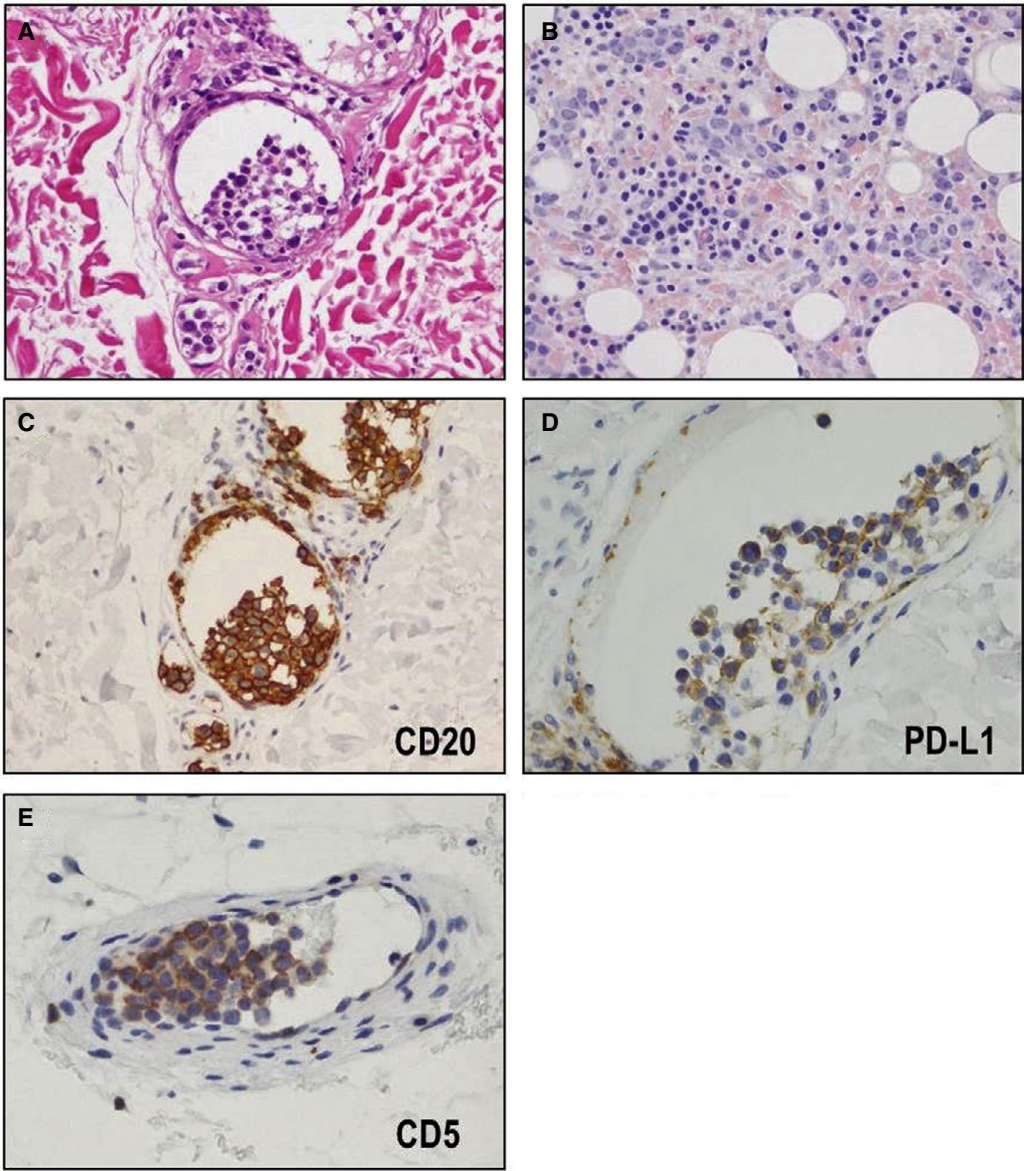
Histological and immunohistochemical features of intravascular large B‐cell lymphoma. Large tumor cells were identified in (A) the capillaries of skin (HE × 400) and (B) bone marrow (HE × 400). Immunohistochemically, these large cells were uniformly positive for (C) CD20 (anti‐CD20 antibody, ×400), (D) 35% (12/34) showed positivity for PD‐L1 (anti PD‐L1 antibody, ×400), and (E) 38% (10/26) showed positivity for CD5 (anti‐CD5 antibody, ×400)

All samples showed prototypic histopathological features that are well documented for IVLBCL. Large tumor cells were identified in the capillaries in each biopsied site or resected organ (Figure 2A,B). Immunohistochemically, these large cells were uniformly positive for CD20 (Figure 2C) and negative for CD3. Twelve (35%) cases were positive for PD‐L1 (Figure 2D). Among 26 cases tested, 10 (38%) were positive for CD5 (Figure 2E). Among 24 cases evaluated with Hans criteria,^29^ 21 (88%) were categorized as a non‐germinal center B‐cell (GCB) subtype. We found no histological or immunohistochemical differences between the PD‐L1^+^ and PD‐L1^−^ groups.

### 3.3 Therapeutic response and prognosis

Among the 34 cases, 27 were treated with R‐CHOP (combination of rituximab, cyclophosphamide, vincristine, adriamycin, and prednisolone); three patients were treated with R‐THPCOP (combination of rituximab, pirarubicin, cyclophosphamide, vincristine, and prednisolone); and one patient was treated with R‐IVAM (combination of rituximab, ifosfamide, etoposide, cytarabine, and methotrexate). One patient was treated with steroid pulse therapy only, and two patients did not receive therapy. Of the 30 patients who received R‐CHOP or R‐THPCOP, nine additionally received high‐dose methotrexate, and 13 additionally received intrathecal chemotherapy. Of the 31 cases that received systemic chemotherapy, 22 achieved complete responses (CR) and three experienced relapses.

Follow‐up data after the therapy were available for 10 and 19 cases of PD‐L1^+^ group and PD‐L1^−^ group, respectively. The responses to initial treatment of 10 cases of PD‐L1^+^ group were as follows: CR (n = 6), stable disease [SD] (n = 1), progressive disease [PD] (n = 2), and not determined (n = 1). Two of the six cases, who achieved CR, had relapse and died of disease. Eventually, four patients died of disease, two patients were alive with disease, and four patients were alive without disease. Regarding the PD‐L1^−^ group, the responses to initial treatment were as follows: CR (n = 16), partial response (n = 1), and PD (n = 2). Eventually, three patients died of disease, one patient was alive with disease, and 15 patients were alive without disease.

**FIGURE 3:**
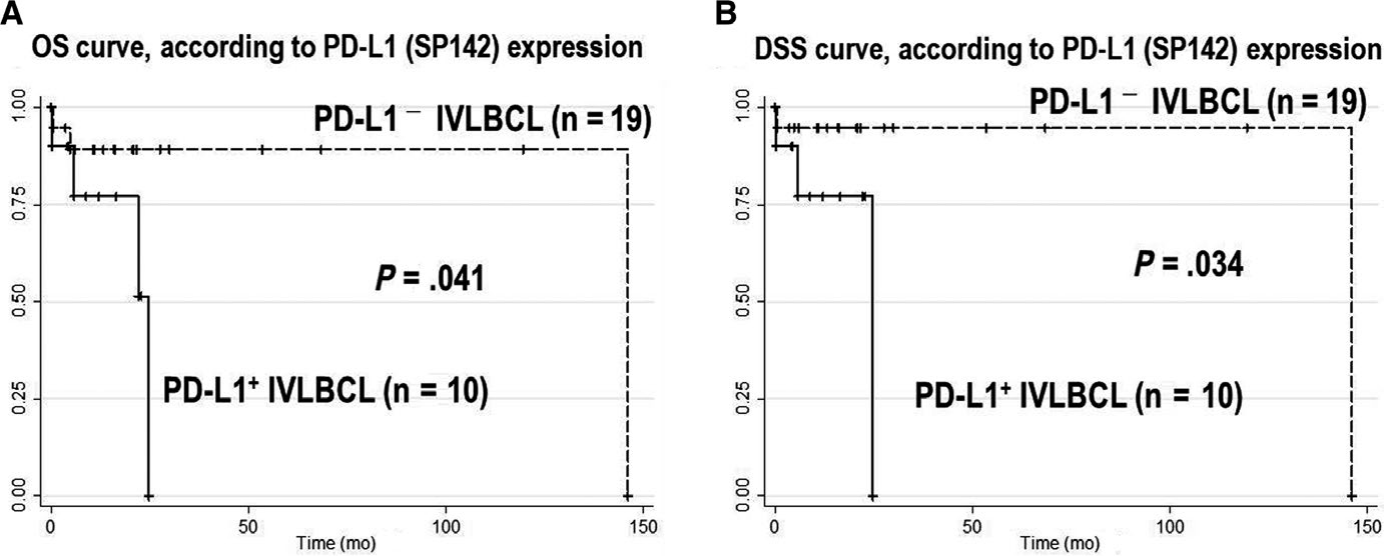
Overall survival (OS) and disease‐specific survival (DSS), according to PD‐L1 expression. The PD‐L1^+^ group showed significantly lower OS (A) and DSS (B) rates compared to the PD‐L1^−^ group (*P* = .041 and .034, respectively)

We compared the outcomes of patients with PD‐L1^+^ and PD‐L1^−^ IVLBCL who received systemic chemotherapy with rituximab. The PD‐L1^+^ group had significantly lower OS and DSS rates compared to the PD‐L1^−^ group (*P* = .041 and .034, respectively; Figure 3).

### 3.4 Comparison of clinicopathological features between PD‐L1^+^ IVLBCL and PD‐L1^+^ eDLBCL

**FIGURE 4:**
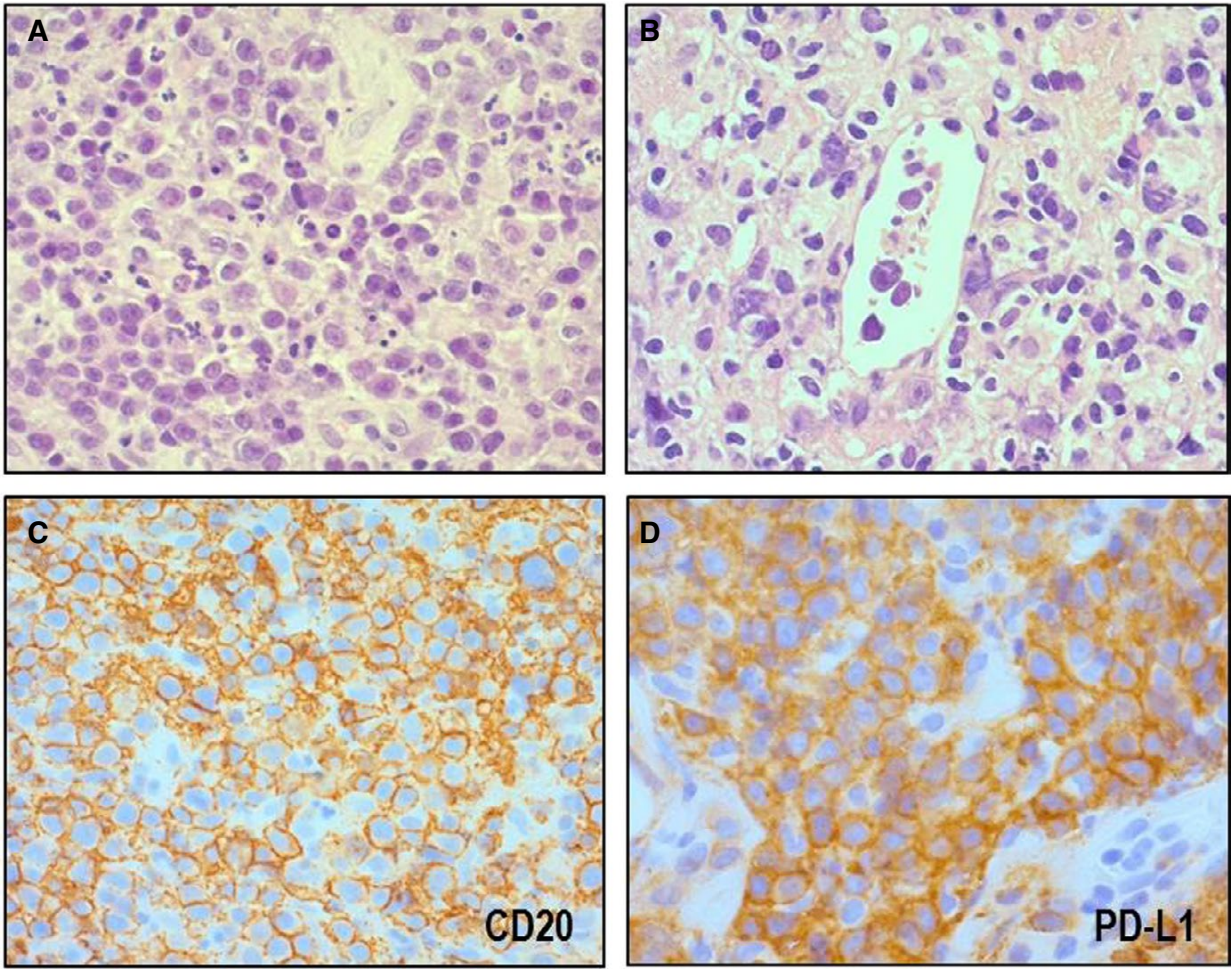
Histological and immunohistochemical features of PD‐L1^+^ extranodal diffuse large B‐cell lymphoma, NOS. Large tumor cells exhibited diffuse proliferation (A, HE × 400). Three of seven cases had intravascular patterns of the tumor cells (B, HE × 400). Immunohistochemically, all cases were consistently positive for CD20 (anti‐CD20 antibody, ×400) and PD‐L1 (anti PD‐L1 antibody, ×400)

As mentioned in the introduction, we speculate that there is possible link between PD‐L1^+^ IVLBCL and PD‐L1^+^ eDLBCL. Therefore, we compared the clinicopathological features between PD‐L1^+^ IVLBCL (n = 12) and PD‐L1^+^ eDLBCL (n = 7). Main involved sites of PD‐L1^+^ eDLBCLs were adrenal gland (n = 2), pelvic cavity (n = 1), kidney (n = 1), spleen (n = 1), bone marrow (n = 1), and ileum (n = 1). Micrographs of representative case are shown in Figure 4. Histologically, all cases exhibited diffuse proliferation of large tumor cells (Figure 4A). Notably, three of seven cases had intravascular patterns of the tumor cells with varying degrees (Figure 4B). Immunohistochemically, all cases were consistently positive for CD20 (Figure 4C) and PD‐L1 (Figure 4D).

**FIGURE 5:**
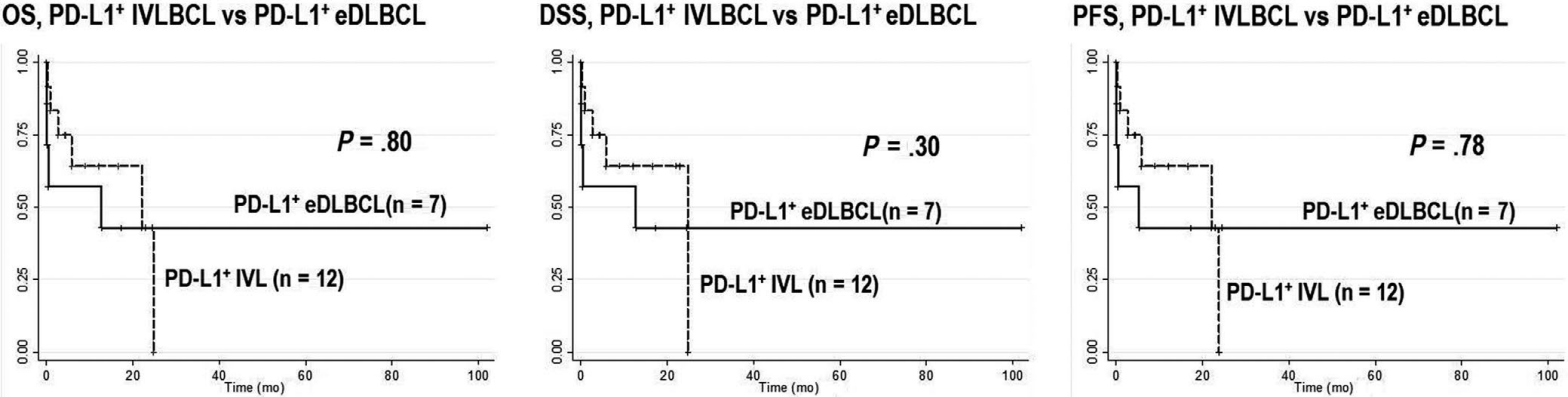
Comparison of overall survival (OS), disease‐specific survival (DSS), and progression‐free survival (PFS) between PD‐L1^+^ intravascular large B‐cell lymphoma (IVLBCL) and PD‐L1^+^ extranodal diffuse large B‐cell lymphoma, NOS (eDLBCL). None of the OS, DSS, and PFS rates were significantly different between PD‐L1^+^ IVLBCL and eDLBCL

Table 2 summarizes the results of comparison between the two groups. There were no significant differences in clinicopathological parameters. We also compared the outcomes between patients with PD‐L1^+^ IVLBCL and PD‐L1^+^ eDLBCL. None of the OS, DSS, and PFS rates were significantly different between the two (Figure 5).

**TABLE 2:** Clinical and phenotypic characteristics of patients with PD‐L1^+^ IVLBCL and PD‐L1^+^ eDLBCL

Variables	PD‐L1^+^ IVLBCL (n = 12)	PD‐L1^+^ eDLBCL (n = 7)	*P*
Sex (male/female)	5/7	4/3	.65
Age, median (range)	74 (51‐81)	72 (59‐84)	.68
Age > 60 y	9/12 (75%)	6/7 (86%)	1.0
Performance status > 1	8/12 (75%)	6/7 (86%)	.60
IPI (HI/H)	10/11 (91%)	5/7 (71%)	.53
Stage III/IV	11/12 (92%)	4/7 (57%)	.12
plt < 14 × 10^4^/μL	9/11 (82%)	5/6 (83%)	1.0
WBC < 3.5 × 10^3^/μL	4/11 (36%)	3/7 (43%)	1.0
Alb < 3.0 g/dL	8/11 (73%)	3/6 (50%)	.59
CRP > 1.0 mg/dL	11/11 (100%)	3/5 (60%)	.083
sIL‐2R > normal	11/12 (92%)	7/7 (100%)	1.0
LDH > normal	11/11 (100%)	6/7 (94%)	.39
B symptoms	8/12 (67%)	5/7 (71%)	1.0
Hepatomegaly	2/12 (8.3%)	1/7 (14%)	1.0
Splenomegaly	7/12 (58%)	2/7 (29%)	.35
CD5 positivity	2/7 (29%)	2/7 (29%)	1.0
COO (GCB/non‐GCB subtype)	1/6	0/7	1.0

Abbreviations: Alb, albumin; CNS, central nervous system; COO, cell of origin; CRP, C‐reactive protein; GCB, germinal center B cell; H, high; Hb, hemoglobin; HI, high‐intermediate; HPS, hemophagocytic syndrome; IPI, international prognostic index; LDH, lactate dehydrogenase; plt, platelet; sIL‐2R, soluble interleukin‐2 receptor; WBC, white blood cell.

## 4 DISCUSSION

In the past few years, neoplastic PD‐L1 expression in malignant lymphoma has increasingly gained attention in the English literature. Generally, studies have indicated that neoplastic PD‐L1 expression is a promising poor prognostic indicator, because it is often associated with adverse clinicopathological parameters in patients.^17^, ^18^, ^30^, ^31^, ^32^, ^33^, ^34^, ^35^, ^36^ However, few reports have focused on this issue in IVLBCL, and those were case reports on a single patient or a short series.^17^, ^25^, ^26^, ^27^, ^37^ Here, we described 34 cases of IVLBCL, which conformed to the clinicopathological picture that has been well documented for this particular disease.

The present study found that neoplastic PD‐L1 expression was detected in 12/34 (35%) cases. Notably, among patients treated with multi‐agent chemotherapy that included rituximab, those with PD‐L1^+^ disease displayed significantly lower OS and DSS rates than those with PD‐L1^−^ disease, despite the younger onset age in the former group. This worse prognosis might be due to mechanisms of immune evasion. These mechanisms have been described previously in PD‐L1^+^ tumor cells. The PD‐1/PD‐L1 pathway induces apoptosis of tumor‐specific CD8^+^ cytotoxic T lymphocytes, which allows tumor cells to escape tumor‐specific and pathogen‐specific immunity mediated by T cells.^10^ Consequently, this mechanism might promote tumor development in IVLBCL. In recent studies, immune check‐point inhibitors have been considered as good agents in treating relapsed or refractory lymphomas.^11^, ^13^, ^15^ In particular, Nayak et al^15^ reported that a PD‐1 blockade with nivolumab was effective in treating relapsed or refractory primary CNS lymphomas. These lymphomas are characterized by frequent copy‐number alterations of 9p24.1/PD‐L1/PD‐L2 and increased PD‐L1 expression.^38^ Moreover, Hishikawa et al reviewed 12 unique cases with coexisting primary CNS lymphoma and IVLBCL,^39^ and Imai et al described the overlapping clinicopathological features of primary CNS lymphoma and IVLBCL in Japanese patients.^40^ Those reports suggested that these two entities might be similar in some pathogen pathways. Taken together, we suggest that patients with PD‐L1^+^ IVLBCL might be good candidates for clinical trials on novel immune check‐point inhibitors in the future.

According to previous reports, 10%‐30% of DLBCL cases expressed PD‐L1 in tumor cells. PD‐L1 expression was associated with a non‐GCB subtype, based on Hans algorithm and EBV positivity.^17^, ^30^, ^41^ Several studies have highlighted mechanisms which cause PD‐L1 overexpression in tumor cells of DLBCL including genetic abnormality (gains, amplifications, or translocations) of the PD‐L1 and PD‐L2. These alterations have affected around 20% of DLBCL cases.^31^, ^42^ They caused direct activation of the PD‐L1 promoter and bring PD‐L1 overexpression. Activation of the JAK/STAT and NF‐κβ pathways is another possible mechanism that might induce PD‐L1 expression. According to previous reports, it has been suggested that PD‐L1 overexpression correlated with the activation of JAK/STAT and NF‐κβ pathways in lymphoid malignancies.^34^, ^36^, ^43^, ^44^, ^45^ Among non‐GCB‐type DLBCL cases, about 30%^46^, ^47^, ^48^, ^49^ harbor *MYD88* mutations, which induce the activation of NF‐κβ and JAK/STAT pathways and cause PD‐L1 overexpression.^46^, ^50^ The present and previous studies have revealed that around 40% of IVLBCL cases were positive for PD‐L1 in tumor cells. This proportion was higher than that reported for DLBCL,^17^, ^25^ but it was consistent with the fact that the majority of IVLBCL cases were the non‐GCB subtype. In the present series, 88% (21/24) of the studied cases showed phenotype of non‐GCB subtype.

Recently, Schrader et al reported that *MYD88* and *CD79B* mutations were detected in 44% and 36% of IVLBCL, respectively.^51^ Both the *MYD88* and *CD79B* mutations induce the activation of the NF‐κβ pathway. Thus, frequent *MYD88* and *CD79B* mutations might explain the elevated PD‐L1 expression rate in IVLBCL. Further investigations are required to unravel the mechanism of PD‐L1 expression in IVLBCL.

We very recently described the clinicopathological findings of two cases of PD‐L1^+^ IVLBCL and four cases of PD‐L1^+^ eDLBCL. We found overlapping features between the two. The PD‐L1^+^ eDLBCL cases showed frequent intravascular patterns, and all of them exclusively affected extranodal sites with no nodal lesions during their entire clinical courses. Therefore, we suggested that PD‐L1^+^ IVLBCL and PD‐L1^+^ eDLBCL might be categorized into one proposed entity, immune evasion‐related extranodal large B‐cell lymphoma. The present study further supports this idea. We highlighted in a larger cohort that clinicopathological characteristics of PD‐L1^+^ IVLBCL and PD‐L1^+^ eDLBCL did not show a significant difference. In addition, none of the OS, DSS, and PFS rates were significantly different between the two. Therefore, we confirmed that PD‐L1^+^ IVLBCL and PD‐L1^+^ eDLBCL may be regarded as one entity called immune evasion‐related extranodal large B‐cell lymphoma.

In summary, we found that PD‐L1 immunohistochemistry could distinguish two prognostically different groups of patients with IVLBCL. The PD‐L1^+^ group had significantly lower survival rates compared to the PD‐L1^−^ group. The PD‐L1^+^ IVLBCL group also had a significantly lower age distribution and a lower frequency of patients older than 60 years, compared to the PD‐L1^−^ group. We speculate that the worse prognosis of the PD‐L1^+^ group was caused by immune evasion mechanisms, which are linked to PD‐L1 expression. Therefore, PD‐L1^+^ IVLBCL cases might be regarded as good candidates for targeted immunotherapy. We also highlighted the overlapping features of PD‐L1^+^ IVLBCL and PD‐L1^+^ eDLBCL and suggest that they should be regarded as one entity, immune evasion‐related extranodal large B‐cell lymphoma.

## CONFLICT OF INTEREST

The authors declare no competing financial interests.

## AUTHOR CONTRIBUTIONS

YS and SN: proposed the study, reviewed the histopathology of all tumors, interpreted the result of the immunostaining, collected the data, and wrote the paper. KK, KM, AS, EI, SS, KS, SM, TT, and SK: collected the data and revised the manuscript. AS: reviewed the histopathology of all tumors, interpreted the result of the immunostaining, collected the data, and wrote the paper.

## Supporting information

Table S1

## Data Availability

The data used to support the findings of this study are available from the corresponding author upon request.
