# Supplementary material for: PD‐L1 (SP142) expression in neoplastic cells predicts a poor prognosis for patients with intravascular large B‐cell lymphoma treated with rituximab‐based multi‐agent chemotherapy
Source: Cancer Med. 2020 May 5;9(13):4768–76. doi: 10.1002/cam4.3104 (PMC7333862; doi:10.1002/cam4.3104)
Supplement: Supplementary file 1 — Table S1 [file CAM4-9-4768-s001.docx]

Clinical features of IVL

| **Case** | **Age** | **Sex** | **PS** | **IPI** | **PD-L1** | **Cytopenia** | **HPS** | **Hepato splenomegaly** | **Respiratory  symp** | **CNS  symp** | **Variant** | **Treatment** | **Res** | **Outcome (months)** |
| --- | --- | --- | --- | --- | --- | --- | --- | --- | --- | --- | --- | --- | --- | --- |
| 1 | 51 | F | 3 | H | + | + | na | + | － | + | HPS | No | na | DOD 0.9 |
| 2 | 74 | F | 4 | H | + | － | na | － | － | + | Classic | Steroid Pulse | na | DOD 2.7 |
| 3 | 72 | F | 3 | H | － | + | na | － | － | － | HPS | R-CHOP+IT | CR | ANED 11 |
| 4 | 76 | M | 4 | H | + | + | na | － | + | － | HPS | R-CHOP | CR | ANED 8.9 |
| 5 | 80 | F | na | na | － | na | na | na | na | na | und | R-CHOP | CR | DOD 146 |
| 6 | 74 | F | 1 | H | － | + | － | + | － | na | HPS | R-CHOP | CR | ANED 54 |
| 7 | 67 | F | 0 | H | + | + | － | + | － | － | HPS | R-CHOP | CR | ANED 17 |
| 8 | 80 | M | 2 | H | － | + | － | － | － | + | HPS | R-THPCOP+IT | CR | ANED 21 |
| 9 | 79 | M | 3 | H | － | + | － | － | － | + | HPS | R-THPCOP | CR | ANED 30 |
| 10 | 74 | F | 4 | na | + | na | － | － | － | + | und | R-THPCOP | SD | AWD 4.2 |
| 11 | 81 | F | 3 | H | － | + | － | － | － | + | HPS | No | na | DOD 1.3 |
| 12 | 75 | M | 2 | H | + | + | － | － | － | + | HPS | R-CHOP+IT | CR | dead 22 |
| 13 | 70 | M | 3 | H | － | + | － | na | na | na | HPS | R-CHOP | PD | dead 4.7 |
| 14 | 57 | F | 0 | HI | + | + | － | + | － | － | HPS | R-CHOP+R-HDMTX | CR | ANED 23 |
| 15 | 74 | F | 4 | H | + | + | － | + | － | + | HPS | IVAM+R | PD | DOD 0.3 |
| 16 | 71 | F | 1 | H | － | + | － | － | + | + | HPS | R-CHOP+R-HDMTX | CR | ANED 22 |
| 17 | 67 | M | 0 | H | － | + | na | － | na | － | HPS | R-CHOP+R-HDMTX | CR | ANED 21 |
| 18 | 73 | F | 4 | H | － | + | － | + | + | + | HPS | R-CHOP+R-HDMTX | CR | ANED 16 |
| 19 | 72 | F | 3 | H | － | + | － | － | + | － | HPS | R-CHOP+IT | CR | ANED 16 |
| 20 | 83 | F | 3 | H | － | + | － | + | + | + | HPS | R-CHOP+IT | CR | ANED 3.6 |
| 21 | 81 | M | 4 | H | + | + | － | + | － | + | HPS | R-CHOP+IT | CR | ANED 12 |
| 22 | 61 | M | 0 | H | + | + | + | + | － | － | HPS | R-CHOP | PD | DOD 5.8 |
| 23 | 75 | M | 1 | H | － | + | － | － | － | － | HPS | R-CHOP+  R-HDMTX+IT | CR | ANED 6.0 |
| 24 | 76 | M | 2 | H | － | + | － | + | na | － | HPS | R-CHOP+  R-HDMTX+IT | CR | AWD 12 |
| 25 | 80 | F | 4 | H | － | + | － | + | + | － | HPS | R-CHOP | PD | DOD 0.6 |
| 26 | 73 | F | 3 | H | － | + | － | － | + | － | HPS | R-CHOP+  R-HDMTX+IT | CR | ANED 68 |
| 27 | 64 | M | 4 | H | － | － | － | － | + | － | Classic | R-CHOP | CR | ANED 120 |
| 28 | 86 | M | 3 | H | － | + | + | + | + | － | HPS | R-CHOP | na | na |
| 29 | 73 | M | 4 | H | － | + | － | － | + | － | HPS | R-CHOP+R-HDMTX | CR | ANED 28 |
| 30 | 77 | M | 2 | H | + | + | － | + | － | － | HPS | R-CHOP+HDMTX | CR | DOD 25 |
| 31 | 80 | M | 0 | LI | － | + | － | + | － | + | HPS | R-CHOP+IT | CR | AWD 11 |
| 32 | 81 | M | 4 | H | － | + | + | na | + | + | HPS | R-CHOP+IT | na | na |
| 33 | 75 | F | 2 | H | － | + | na | + | － | － | HPS | R-CHOP+IT | PR | AWD 4.8 |
| 34 | 55 | F | 0 | LI | + | － | － | － | － | － | Classic | R-CHOP+IT |  | AWD 4.4 |
